# Diattenuation and retardance signature of plasmonic gold nanorods in turbid media revealed by Mueller matrix polarimetry

**DOI:** 10.1038/s41598-021-99430-6

**Published:** 2021-10-08

**Authors:** Subir Kumar Ray, Nirmalya Ghosh, Alex Vitkin

**Affiliations:** 1 Division of Biophysics and Bioimaging, Princess Margaret Research Institute, Toronto, Canada; 2 Department of Medical Biophysics, University of Toronto, Toronto, Canada; 3 Indian Institute of Science Education and Research (IISER) Kolkata, Mohanpur, 741246 India; 4 Department of Radiation Oncology, University of Toronto, Toronto, Canada

**Keywords:** Biophysics, Nanoscience and technology, Optics and photonics, Physics

## Abstract

Plasmonic gold nanorods (GNRs) are finding increasing use in biomedicine due to their unique electromagnetic properties, optical contrast enhancement and biocompatibility; they also show promise as polarization contrast agents. However, quantification of their polarization-enhancing properties within heterogeneous turbid media remains challenging. We report on polarization response in controlled tissue phantoms consisting of dielectric microsphere scatterers with varying admixtures of GRNs. Experimental Mueller matrix measurements and polarization sensitive Monte-Carlo simulations show excellent agreement. Despite the GNRs’ 3D random orientation and distribution in the strong multiply scattering background, significant linear diattenuation and retardance were observed. These exclusive measurable characteristics of GNRs suggest their potential uses as contrast enhancers for polarimetric assessment of turbid biological tissue.

## Introduction

Surface plasmon resonance (SPR) is the collective oscillation mode of free conduction electrons at the metal–dielectric interface irradiated by an electromagnetic (EM) wave, causing strong field enhancement and localization at the interface. SPR decays rapidly with distance, enabling precise localization of the process and furnishing a potentially effective tool for imaging, sensing, and optical manipulations^1–4^. For example, light incident onto a medium containing metallic nanoparticles causes excitation of localized surface plasmons, affecting the intensity and the spectral content of the reflected or transmitted light and thereby enhancing optical contrast^5^. Gold nanoparticles (GNPs) exhibit strong localized SPR effect and have thus been actively explored in biology and medicine^6–8^. Their strong confinement of EM waves and biocompatibility has spurred their use as multifunctional probes for molecular rulers^9^, single molecule detection^10^, photoactive drug delivery^11^, photodynamic therapy^12^, photothermal treatments^13^, stem cells targeting^14^, tumour and stromal microenvironment imaging^15^, and optical contrast enhancement^16^. In this context, plasmonic gold nanospheres have not proven particularly useful because their plasmon resonance effects occur at shorter wavelengths, which owing to basic tissue optics limits their biomedical use^17^. However, plasmonic gold nanorods (GNRs) have been found to be more useful due to their geometry and aspect ratio dependent tunable plasmon mode which falls within the visible and near infrared spectral regime suitable for biomedical imaging^6–8^. Several studies have therefore focused on GNR delivery mechanism, their interaction with biological media, and their photoactivity^12–17^.

The two different polarizabilities along the two orthogonal directions of the plasmonic GNRs allow free conduction electrons to oscillate and resonate along the radial (transverse mode) and the axial (longitudinal mode) direction. These produce transverse and longitudinal surface plasmon resonances with different resonance wavelengths within the biologically relevant spectral range. As a result, GNRs exhibit large differences in both intensity and phase of the scattered light depending on its incident polarization. This manifests as extremely large and resonance-enhanced polarization effects of the GNRs^18,19^. These intriguing polarization effects are manifested as (1) differential attenuations (absorption and scattering) between two orthogonal linear polarization states, (2) difference in phase between two orthogonal linear polarization states (retardance) and (3) incident-polarization-state-dependent degree of polarization (*DOP*). Importantly, these resonance-enhanced linear diattenuation and retardance effects are also wavelength tunable through the size and aspect ratio of the GNRs and can generally be observed even from a randomly dispersed collection of the GNRs, making them a promising candidate for polarization-optimized bioimaging.

In general, propagation of polarized light through optically turbid biological medium results in depolarization, diattenuation, and retardance effects. Multiple scattering is often the primary source of depolarization, although other inhomogeneities—for example, randomly oriented birefringent domains—also contribute to polarization loss^20,21^. Whatever the mechanism, the unique scattering asymmetries of GNRs imbedded in such a random inhomogeneous medium may get completely washed out due to strong multiple scattering background. Additionally, random orientations of GNRs invariably introduce stochastic variation of polarization states, increasing decoherence and entropy, thus leading to further depolarization and weakening of the intrinsic diattenuation and retardance effects.

It has previously been shown that for a *single isolated* plasmonic GNR, the differential scattering cross-section between horizontally and vertically polarized light approaches unity in the vicinity of GNR’s longitudinal plasmon resonance (and occurs at longer wavelength than the transverse plasmon resonance)^19,22,23^. This suggests that isolated plasmonic GNRs can act as a nearly perfect linear diattenuator^19,23^. However, this idealized scenario does not account for (1) large background tissue depolarization, and (2) the potential loss of GNRs’ polarization signatures due to their random 3D spatial orientation. It is thus crucial to understand detailed polarization response from random distributions of multiple GNRs inside heterogeneous media such as biological systems. There have only been a few works on the use of depolarization mapping approach in this context^23,24^. For example, Lippok et al. has illustrated the diffusion and distribution of GNRs in biologically relevant scenarios^24^. The authors described the statistics of polarization states using the density matrix formalism and the Mueller matrix-derived depolarization coefficient. However, such advanced analysis may be limited to gated detection of ballistic-like photons scenarios, necessitating the use of complex partially coherent imaging systems for its practical realization. The MM derived polarimetry parameters such as diattenuation, retardance and (importantly) its orientation provide anisotropic organizational information on the tissue collagen fibre network^25^. Such polarization parameters may serve as additional metrics for characterizing various biological tissues and their pathologies, for example with significant connective tissue (stromal) involvement such as cancer development^26^.

We therefore pursue a more direct polarimetric study of turbid media based on optical polarization signatures (linear diattenuation and retardance) of plasmonic GNRs derived from Mueller matrix measurements, in carefully designed tissue phantoms comprised of scattering polystyrene microspheres and GNRs. The experimental results are supported by numerical simulations of T-matrix-modified polarization sensitive Monte-Carlo (PSMC) model. Importantly, both theory and experiments reveal significant magnitude of linear diattenuation and retardance effects stemming from the longitudinal and transverse plasmon resonances of the GNRs, despite their random orientation and the presence of strong multiple scattering backgrounds.

## Theory

### Modelling single scattering from plasmonic Au GNRs

Gustav Mie in his celebrated work^27^ showed that the expansion of incident and scattered electric fields in vector-spherical harmonic functions yields an exact solution for light scattering by *spherical particles*. In the far field, this approach relates the incident and the scattered electric fields through the Jones formalism by $E_{s} = {\mathbf{J}}E_{i}$ where ${\mathbf{J}}$ is the 2 × 2 amplitude scattering Jones matrix, $E_{i}$ and $E_{s}$ are the incident and the scattered electric fields, respectively. Since the Mie theory is based on spherical symmetry, various extensions to *non-spherical particles* have also been proposed^28–33^. Among these, the T-matrix method has been the most widely used for modeling particles with an axis of symmetry, such as ellipsoids, spheroids, and cylinders^31^. Using the T-matrix framework, the incident, and the scattered fields from a long cylindrical scattered (length $\gg$ diameter) can be expressed as^30^:

1 $$\left( {\begin{array}{*{20}c} {E_{\parallel s} } \\ {E_{ \bot s} } \\ \end{array} } \right) = e^{i3\pi /4} \sqrt {\frac{2}{\pi kr\sin \xi }} e^{ik(r\sin \xi - z\cos \xi )} \left( {\begin{array}{*{20}c} {T^{11} } & {T^{12} } \\ {T^{21} } & {T^{22} } \\ \end{array} } \right)\left( {\begin{array}{*{20}c} {E_{\parallel i} } \\ {E_{ \bot i} } \\ \end{array} } \right)$$

where $E_{\parallel i}$, $E_{ \bot i}$ and $E_{\parallel s}$, $E_{ \bot s}$ represent incident and the scattered electrical fields polarized parallel and perpendicular to the scattering plane, $r$ and $z$ are the cylindrical polar coordinates, $k$ is the wave number, $\xi$ is the oblique incident angle between the incident light and the longitudinal axis of the cylinder. The elements $T^{11} ,T^{12} ,T^{21}$ and $T^{22}$ form the amplitude scattering matrix (“the T matrix”). However, slight deviation from ideal finite cylindrical shape of GNRs may results in significant changes in the scattering, absorption, and extinction properties. To obtain a more realistic model for non-ideal finite cylinders, we incorporated a more generalized T-matrix model^33^ into our PSMC simulation engine. Specifically, this model allows one to incorporate five modified parameters for individual GNRs: length, central diameter, end-cap diameter, end-cap elliptical thickness and the shape of the generating line between the end caps. The details of these modifications to Eq. (1) are described in Supplemental Materials. The resultant differential scattering, absorption, and extinction cross-sections of individual GNRs were then used to calculate 3D-orientation-averaged scattering, absorption and extinction cross-sections for multiple GNRs, as described in “Simulation of polarized light propagation in turbid media containing dielectric microspheres and GNRs” section below.

### Monte Carlo modeling of polarized light propagation in multiply scattering tissue-like turbid media containing dielectric microspheres

**Figure 1:**
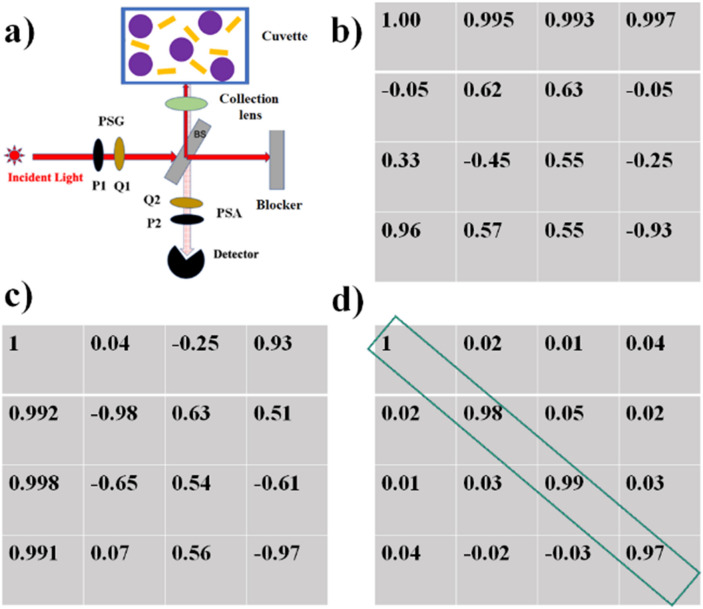
Experimental polarimetry set up and system calibration for exact backscattering configuration. (**a**) Schematic diagram of the experimental polarimetry system for measurements in exact backscattering configuration, comprised of an excitation laser (λ = 633 nm), a polarization state generator unit (PSG: rotating linear polarizer P1 and quarter wave plate Q1), a polarization state analyzer unit (PSA: rotating quarter wave plate Q2 and linear polarizer P2), beam splitter (BS), collection lenses and a photodetector. Actual experimental form of the (**b**) PSG matrix W and (**c**) PSA matrix A. (**d**) The experimental Mueller matrix from a cuvette filled with distilled water resembles an identity matrix as expected, with individual elements exhibiting an error $\le 0.05$.

A previously developed and validated polarization sensitive Monte Carlo (“polMC”) model^20,34^ was used to track and record the polarization status of photon packets propagating in a turbid medium containing dielectric polystyrene microspheres and gold nanoparticles having the shape of finite cylinders (GNRs). For the turbid media comprising of aqueous suspension of dielectric microspheres-only, position and propagation direction of individual photon packets were tracked along with the polarization information using the Stokes vector. The final values of the different Stokes vector elements were computed as the sum of all the appropriate photon subpopulations exiting the sample. For this study, the photon collection geometry in the backscattering direction was chosen to have a detection area of 1 mm^2^ and an acceptance angle of 10°. These selected parameters mimic our experimental polarimetry configuration (Fig. 1a). The refractive index of the background medium was taken as 1.334 to represent water with no absorption. Spherical polystyrene microspheres with diameters $0.42,$ $0.65$ and $0.92$ μm and refractive index $1.59$ were used as dielectric scatterers. The photon wavelength was 632.8 nm. Using standard Mie theory calculations for spherical dielectric particles^27,30^, the scattering efficiency $Q$ and scattering anisotropy parameter ($g$, the average cosine of scattering angle) were calculated. The former value was used to scale the microsphere concentration to yield the medium scattering coefficient $\upmu_{s} = 102\,\hbox{cm}^{ - 1}$, which is typical of mammalian tissues in the “optical window” spectral regime. The simulations were carried out with $10^{9}$ photons. The incident polarizations were horizontal, vertical, + 45°, − 45°, left and right circular states using the standard Stokes vector description. The Stokes vector elements of the backscattered light emerging in the 10° cone centered on the backscattering direction were recorded for each of the input polarization states. The Mueller matrix of the medium was constructed by performing standard algebraic manipulations using the recorded output Stokes parameters for each respective input state^20,34^.

### Simulation of polarized light propagation in turbid media containing dielectric microspheres and GNRs

The case of randomly oriented GNRs within the multiply scattering dielectric medium containing polystyrene microspheres were simulated next. First, unlike the symmetrical case of the microspheres, the scattering and polarization effects of light interactions with GNRs are dependent on the orientation of the latter. That is, the scattering matrix of finite cylindrically shaped GNRs varies with the angle $\xi$ between the direction of the incident photon and the orientation of GNR’s cylindrical axis. To address this issue, we resort to the single scattering T-matrix formalism for GNRs (“Modelling single scattering from plasmonic Au GNRs” section above) to study polarization preservation in a collection of randomly oriented 3-dimensional distributions of finite GNRs inside a turbid medium. For a monodispersed (same sized) ensemble of randomly oriented GNRs with constant mass-volume concentration of gold $c_{g}$ per unit water suspension volume, the extinction coefficient is given by^33^

2 $$A_{ext} = \frac{{N\left\langle {C_{ext} } \right\rangle l}}{\ln 10}\frac{{3c_{g} l}}{{4\ln 10\rho_{g} }}\frac{{\left\langle {Q_{ext} } \right\rangle }}{{a_{0} }}$$

Here, $l$ is the water medium thickness (1-cm-thick glass cuvette in our experiments), $\left\langle {C_{ext} } \right\rangle = \pi a_{0}^{2} \left\langle {Q_{ext} } \right\rangle$ is the extinction cross section, the averaging over GNR orientations is represented by the angular brackets, $\rho_{g} \left( {19.3\,\hbox{gm/cc}} \right)$ is the density of gold, $N = c_{g} /V\rho_{g}$ is the “numerical concentrations” (number of GNRs per unit volume of water suspension), $a_{0} = \left( {{{3V} \mathord{\left/ {\vphantom {{3V} {4\pi }}} \right. \kern-\nulldelimiterspace} {4\pi }}} \right)^{1/3}$ is the equi-volume radius, and $V$ is the volume of all the GNRs sampled by light in the water suspension.

T-matrix analytical solutions for orientation-averaged scattering and extinction cross-sections are then given by^33^

3 $$\begin{aligned} \left\langle {C_{sca} } \right\rangle & = \frac{2\pi }{{k^{2} }}\sum\limits_{{nn^{\prime } ,mm^{\prime } ,ij}}^{\infty ,n,2} {\left| {T_{{mn,m^{\prime } n^{\prime } }}^{ij} } \right|^{2} } \\ \left\langle {C_{sca} } \right\rangle & = \frac{2\pi }{{k^{2} }}{\text{Re}} \sum\limits_{n = 1,m = - n,i = 1}^{\infty ,n,2} {T_{mn,nmn}^{ij} } \\ \end{aligned}$$

where $\sum\nolimits_{nn^{\prime},mm^{\prime},ij}^{\infty ,n,2} {\left| {T_{mn,m^{\prime}n^{\prime}}^{ij} } \right|}^{2} = \sum\nolimits_{nn^{\prime},mm^{\prime}}^{\infty ,n,2} {\left| {p_{mn} } \right|}^{2} + \left| {q_{mn} } \right|^{2} ;$ ${\text{Re}} \sum\nolimits_{n = 1,m = - n,i = 1}^{\infty ,n,2} {T_{mn,nmn}^{ij} } = {\text{Re}} \sum\nolimits_{n = 1,m = - n}^{\infty ,n,2} {\left[ {a_{mn} \left( {p_{mn} } \right)^{*} + b_{mn} \left( {q_{mn} } \right)^{*} } \right]} ;$ $a_{mn} ,b_{mn}$ and $P_{mn} ,q_{mn}$ are the expansion coefficients of the incident and scattered-field waves respectively. These two expansion coefficients are related as

3A $$\begin{aligned} p_{mn} & = \sum\limits_{{n^{\prime } = 1}}^{\infty } {\sum\limits_{{m^{\prime } = - n^{\prime } }}^{{n^{\prime } }} {\left( {T_{{mnm^{\prime } n^{\prime } }}^{11} a_{{m^{\prime } n^{\prime } }} + T_{{mnm^{\prime } n^{\prime } }}^{12} b_{{m^{\prime } n^{\prime } }} } \right)} } \\ q_{mn} & = \sum\limits_{{n^{\prime } = 1}}^{\infty } {\sum\limits_{{m^{\prime } = - n^{\prime } }}^{{n^{\prime } }} {\left( {T_{{mnm^{\prime } n^{\prime } }}^{21} a_{{m^{\prime } n^{\prime } }} + T_{{mnm^{\prime } n^{\prime } }}^{22} b_{{m^{\prime } n^{\prime } }} } \right)} } \\ \end{aligned}$$

In the presence of both microspheres and GNRs, the generalized extinction of a polydisperse ensemble consisting of $n_{R}$ and $n_{S}$ (number fractions of GNRs and microspheres, respectively) can be obtained from Eqs. (2) and (3) as

4 $$A_{ext} = \frac{{c_{g} }}{{\ln 10\rho_{g} V_{t} }}\left[ {W_{R} \sum\limits_{i = 1}^{{n_{S} }} {n_{{R_{i} }} \left\langle {C_{{ext,R_{i} }} } \right\rangle } } \right] + W_{S} \sum\limits_{i = 1}^{{n_{S} }} {n_{{S_{i} }} \left\langle {C_{{ext,S_{i} }} } \right\rangle }$$

Here, $c_{g}$ and $\rho_{g}$ are mass-volume concentration and density of gold, $V_{t}$ is the total volume of scatterers (GNRs and microspheres) per unit suspension volume, and $w_{R/S} = \left( {n_{R/S} } \right)/n$. We have thus replaced the Mie scattering function with Eq. (4) in the PSMC simulation described in “Monte Carlo modeling of polarized light propagation in multiply scattering tissue-like turbid media containing dielectric microspheres” section above. Simulations were carried out for a fixed $W_{S}$ (corresponding to microsphere-induced $\upmu_{S} = 102\,\hbox{cm}^{ - 1}$ value with $0.42\,\upmu \hbox{m}$ diameter microspheres), whereas the values of $w_{R}$ (corresponding to GNR-induced $\upmu_{S}$ in the 16 to 195 cm^−1^ range) were varied. Tracking the position, direction, and Stokes’s vector of each photon packet described above then enables the Mueller matrix elements of the backscattered detected light to be constructed.

### Mueller matrix inverse analysis to quantify depolarization, diattenuation and retardance signatures of GNRs

The general form of a Mueller matrix of an arbitrary sample is given by $M\left( \theta \right)$, where the angular dependence emphasizes that the polarization response varies with the measurement geometry:

5 $$M\left( \theta \right) = \left( {\begin{array}{*{20}c} {M_{11} } & {M_{12} } & {M_{13} } & {M_{14} } \\ {M_{21} } & {M_{22} } & {M_{23} } & {M_{24} } \\ {M_{31} } & {M_{32} } & {M_{33} } & {M_{34} } \\ {M_{41} } & {M_{42} } & {M_{43} } & {M_{44} } \\ \end{array} } \right)$$

The elements of $M$ contain useful polarization information about the interrogated medium in terms of the sample polarization properties, namely, *depolarization*, *retardance* and *diattenuation*. Depolarization $\Delta$ is a quantitative measure of the net decrease in the degree of polarization ($DOP = \frac{{\sqrt {\left( {Q^{2} + U^{2} + V^{2} } \right)} }}{I}$) where $I,Q,U$ and $V$ are the elements of the light’s Stokes vector, arising due to the heterogeneous nature of the scattering medium. The quantity depolarization is usually defined as $\Delta = 1 - DOP$; for example, fully polarized light having net $DOP = 1$ would correspond to no depolarization or $\Delta = 0$, whereas unpolarized light with $DOP = 0$ would imply complete depolarization with $\Delta = 1$. Retardance $R$ is defined by the phase shift between two orthogonal polarization states, arising from the directional asymmetries in the real part of the refractive index of the medium (e.g., in a birefringent crystal or striated biological tissues such as muscle fibers or collagenous connective tissue). Analogously, diattenuation $D$ is defined as the differential attenuation of orthogonal polarization states, which arises due to difference in the imaginary part of the refractive index between orthogonal polarizations. Thus, whereas retardance quantifies difference in phase shifts, diattenuation reports on intensity differences (due to differential absorption and scattering) between orthogonal polarizations.

All the medium polarization properties are contained in the various elements of the Mueller matrix in a complex, interrelated way. Extraction, quantification and unique interpretation of these is possible by mathematically decomposing the Mueller matrix into ‘basis’ metrices of elementary polarization properties. This is not a unique process, and several variants of this decomposition exist with their relative merits extensively discussed in the literature^34–37^. Among the different options, the Lu and Chipman’s Polar Decomposition Method is the most widely used and was also utilized here^37^. The extraction of $D,R$ and $DOP$ parameters via this decomposition approach is further described in the “Supplementary Materials” section.

## Results and discussions

Numerical simulations and experiments were performed in the backscattering configuration on polystyrene microspheres suspensions of water without and with the additions of the GNRs. The utility of the proposed approach was demonstrated by decoupling and quantifying the polarization response of the dielectric background of the optically turbid samples and the important polarization effects of localized surface plasmon resonances (LSPRs) of the GNRs.

Experimental calibration results are presented in Fig. 1, where a schematic of the experimental configuration is shown in Fig. 1a. Corresponding experimentally obtained PSG matrix *W*, PSA matrix *A* and the extracted blank Mueller matrix of the pure-water-filled cuvette are presented in Fig. 1b–d respectively. Each column of the PSG matrix *W* (Fig. 1b) represents a Stokes vector corresponding to the optimized orientation angles $\theta = 35^{o} ,70^{o} ,105^{o}$ and $140^{o}$) of the quarter waveplate *Q1*. For example, first column of Fig. 1b represents the normalized Stokes vector for $\theta = 35^{o}$. Similarly, each row of the PSA matrix *A* in Fig. 1c represents the top row of the combined Mueller matrix of the optical elements forming the PSA. Experimentally calibrated Mueller matrix for a non-depolarizing sample (blank cuvette filled with pure water) shown in Fig. 1d yields a 4 × 4 array that closely resembles an identity matrix with an elemental error $\le 0.05$*,* which may be considered as the elemental accuracy of our Mueller matrix measurement system.

### Polarization dependency of the microsphere phantom system

**Figure 2:**
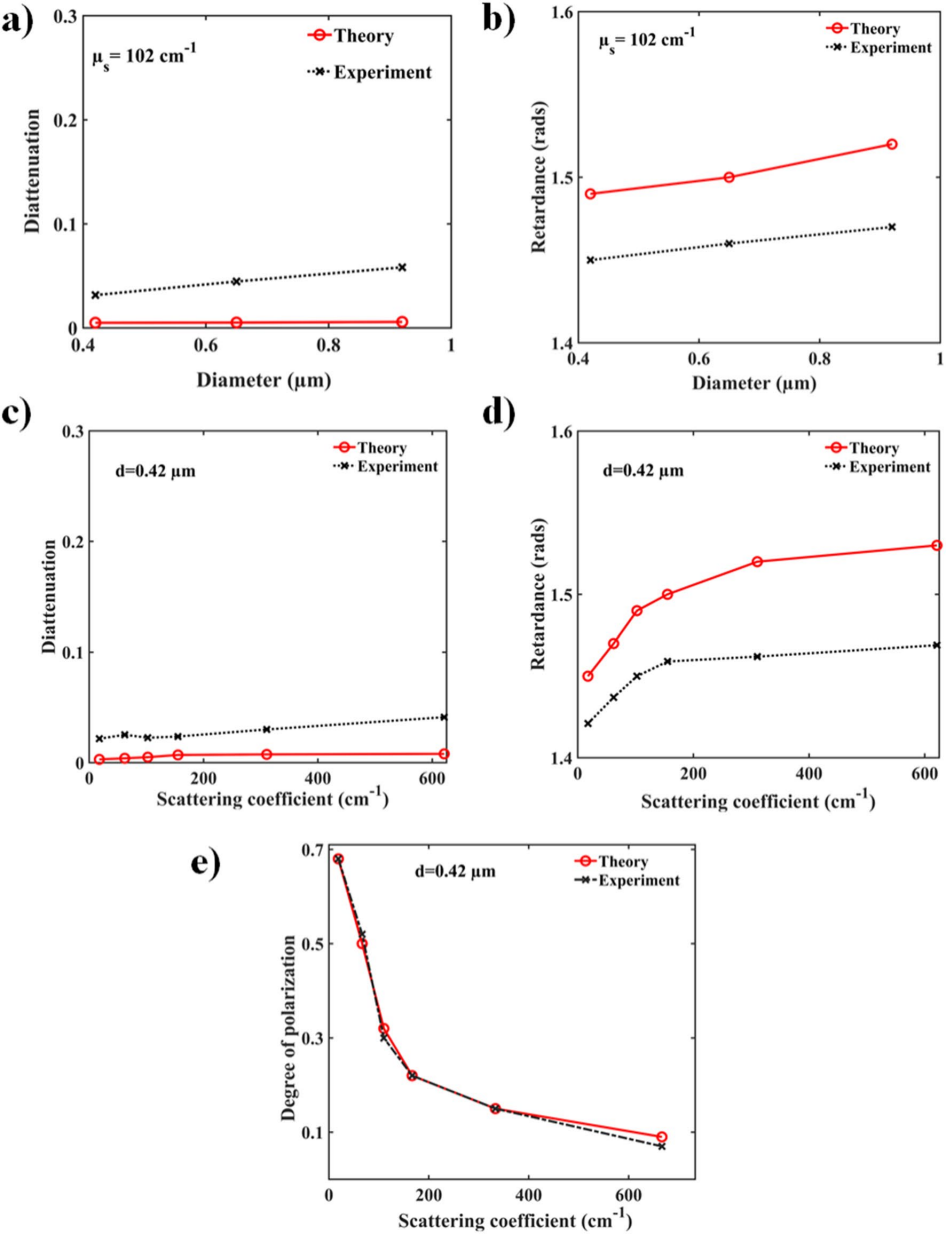
Polarization effects in microspheres-only phantom system. Dependence of the Mueller matrix-derived polarization parameters of a controlled turbid media (microsphere phantom) on the diameter and scattering coefficient. MC-simulated (red symbols) and experimentally obtained (black symbols) variations of the (**a**) diattenuation and (**b**) retardance with microsphere diameters for a fixed scattering coefficient $\upmu_{S} = 102\,\hbox{cm}^{ - 1}$. Near zero diattenuation stems from isotropic nature of the spherical scatterers, and non-zero retardance ($\sim 1.5$ radians) likely arises from the detection geometry effects and particle diameters (for details, see text). The simulated (red symbols) and experimental (black symbols) variations of (**c**) diattenuation and (**d**) retardance with optical turbidity ($\upmu_{S}$ range from 16 to 621 cm^−1^) for a fixed scatterer diameter ($d = 0.42\,\upmu$m) exhibit similar trends of near zero diattenuation and finite retardance (− 1.4 to 1.5 radians). The corresponding variation of the simulated and experimental degree of polarization with turbidity shown in (**e**). *Note*: in Figs. 2 and 3, symbols are experimental and simulations results, and lines are guides for the eye.

Polystyrene microsphere phantom systems of three different diameters ($$0.42,0.65$$ and $$0.92\,\upmu$$m) at varying concentrations were investigated to determine polarization effects in turbid media. Mueller metrices were experimentally recorded from the phantoms and were subsequently analyzed by the Mueller matrix decomposition method to extract and quantify the medium polarimetry parameters (depolarization $$\Delta$$, diattenuation $$D$$ and retardance $$R$$), following the procedure discussed in “Mueller matrix inverse analysis to quantify depolarization, diattenuation and retardance signatures of GNRs” section. Similar analysis was also performed on the corresponding Mueller metrices generated by the PSMC model. The extracted diattenuation and retardance parameters are shown for both approaches in Fig. 2a,b. Linear diattenuation values for three different microsphere diameters obtained from PSMC simulation are observed to be negligible. The corresponding experimental diattenuation values are also quite low, within the typical elemental error ($$\sim 0.05$$) and thus agree well with the results of the simulations. Indeed, the scattering from spherically symmetric dielectric microspheres is not expected to produce any diattenuation effect in the backscattering configuration, as seen both theoretically and experimentally here. However, it is known that scattering from large dielectric spheres (size parameters $$x > 1,$$$$x = 2\pi an/\lambda$$, where $$a$$ is the radius of the scatterer, $$N$$ is its refractive index and $$\lambda$$ is the wavelength of light) can lead to significant linear retardance effects that depend on the particle diameter and detection angle^19,38^. This has been previously attributed to the excitation of the different higher order transverse magnetic ($$TM - a_{n}$$) and transverse electric ($$TM - b_{n}$$) Mie modes of dielectric spheres and their complex interference effects that depend on light’s polarization state^19^. Thus, both the theoretical and experimental Mueller metrices yielded finite linear retardance values, of the order of $$\sim 1.5$$ radians for all three microsphere diameters.

Figure 2c,d summarize the observed trends of scattering-induced linear diattenuation and retardance obtained through PSMC simulations and experiments from varying-turbidity for the 0.42 µm diameter microsphere suspensions. The scattering coefficients ($\upmu_{S}$) were varied from − 16 to 621 cm^−1^. Corresponding changes in the magnitude of diattenuation was in the range $0.0113 - 0.0147$(simulations) and $0.0217 - 0.0412$ (experiment). Similarly, the observed changes in the magnitude of linear retardance spans 1.45–1.53 rads (theory) and 1.42–1.47 rads (experiment). Thus, theory and experiments show good agreement on the behavior of both these important polarization metrics with turbidity. As noted above, while diattenuation is consistently negligible from spherical scatterers, the non-zero magnitude of retardance is influenced by a number of factors like scatterer size, shape and concentration, signal collection angle etc. which affects the interference of the higher order $TM$ and $TE$ scattering modes in a complex way^19,38^.

Polarization preservation in the exact backscattering direction is a well-known effect^20,38^ and is also quite evident from the results presented in Fig. 2e where excellent agreement between theory and experiment is once again seen. We also note that the circular depolarization was consistently observed to be stronger than linear depolarization in our backscattering investigations, both theoretically and experimentally (data not shown). These results agree with previous reports demonstrating that the characteristic length for depolarization of linearly polarized light is considerably higher than circularly polarized light for turbid media comprised of larger sized scatterers (typically for size parameters $x > 1,$ in our case $x \sim 3.3$)^38^. The nonzero values of $DOP$ for large values of scattering coefficients suggest that even for a highly turbid medium, some polarization information is preserved and quantification of polarization parameters in backscattering geometry is possible.

### Polarization dependency of GNRs within a microsphere phantom system

**Figure 3:**
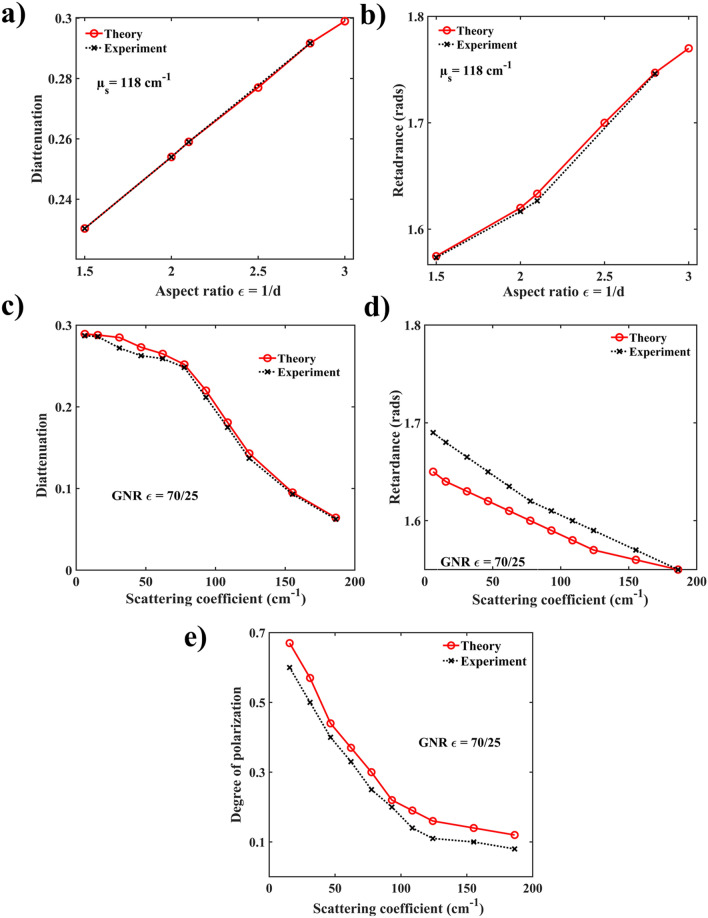
Polarization effects in a composite microsphere + GNRs phantom system. Mueller matrix simulated (red symbols) and experimentally obtained (black symbols) variation of the (**a**) diattenuation and (**b**) retardance with GNR's aspect ratio $\in$ for a fixed scattering coefficient of the composite system($\upmu_{{_{S} }} = 102\,\hbox{cm}^{ - 1}$ due to microspheres plus $16\,\hbox{cm}^{ - 1}$ due to GNRs). Increasing diattenuation and retardance with aspect ratio suggests size-dependent increase of the optically anisotropic nature of GNRs. Simulated (red symbols) and experimental (black symbols) variation of diattenuation and retardance with GNRs concentration in a turbid phantom of fixed microsphere scattering properties (diameter = $0.42\,\upmu$m, $\upmu_{S} = 102\,\hbox{cm}^{ - 1}$). The geometrical parameters of GNRs were length $l = 70\,\hbox{nm}$ diameter $d = 25\,\hbox{nm}$(aspect ratio $\in = 2.8$). Both $D$ and $R$ exhibit significantly higher values (better contrast) at lower nanorod concentrations ($\upmu_{S} = 16\,\hbox{cm}^{ - 1}$), implying strong orthogonal dipolar plasmon polarizabilities even for 3D random orientations of GNRs (for details, see text). The corresponding variation of the simulated and experimental degree of polarization on the GNR concentration shown in (**e**).

We now proceed to investigate the interesting polarization effects due to the presence of plasmonic GNRs in a turbid phantom with fixed microsphere concentration (scattering coefficient $\upmu_{S} = 102\,\hbox{cm}^{ - 1}$). Once again, the Mueller matrix-derived polarization parameters obtained using the polarization sensitive Monte Carlo simulations will be compared to those from experimental Mueller matrix measurements. As noted in “Simulation of polarized light propagation in turbid media containing dielectric microspheres and GNRs” section, we have modeled the orientation-averaged extinction, scattering and absorption cross sections for an ensemble of randomly oriented GNRs with constant mass-volume concentrations of gold per unit volume of water suspension containing microspheres, using Eq. (4). The polarization signature of randomly oriented nanorods was studied by determining all the sixteen scattering Mueller matrix elements $M_{ij}$, which transform the Stokes parameters ($I,Q,U,V$) of the incident light into the corresponding set of scattering Stokes’s parameters. The averaging over random orientations was performed numerically as described in “Simulation of polarized light propagation in turbid media containing dielectric microspheres and GNRs” section by taking the ensemble average of scattering matrix elements. The Mueller matrix-derived diattenuation, retardance and degree of polarization parameters are summarized in Fig. 3. The diameter of the microspheres was kept fixed as 0.42 µm, and geometrical dimensions of GNRs ranged in length $l = 71$ to 105 nm and $d = 25$ to 70 nm. The concentration and the aspect ratios $\left( { \in = l/d} \right)$ of the GNRs were systematically varied to study the corresponding changes in the polarization parameters.

Figure 3 summarizes the results of the polarization characteristics of GNRs in turbid medium. Significant linear diattenuation and retardance effects were observed both in the simulated and in the experimentally recorded Mueller metrices. Specifically, the significant magnitude of diattenuation appears to be a characteristic polarization signature of the GNRs, given low diattenuation observed from the microsphere-only turbid phantoms (Fig. 2a–d). Comparison of the PSMC simulated and experimental Mueller matrix-derived linear diattenuation $D$ and linear retardance $R$ with varying aspect ratio $\in$ are shown in Fig. 3a,b, respectively. The origin of the observed strong linear diattenuation and retardance effects due to scattering from GNRs is worth a brief mention here^19,39^.

These effects arise due to the differences in amplitudes and phases, respectively, of the two orthogonal (longitudinal and transverse) dipolar plasmon polarizabilities of the GNRs. Strong linear diattenuation is thus manifested due to polarization-selective resonance of the two plasmon polarizabilities (longitudinal and transverse plasmon resonances excited by horizontal and vertical linear polarizations respectively) and subsequent differential scattering intensities for the two orthogonal linear polarizations. Such resonance-enhanced linear diattenuation in plasmonic nanorods is known to exhibit its maximum magnitude near the peak wavelength of the longitudinal plasmon resonance^19,39^. Linear retardance, on the other hand, arises due to the resonance-enhanced phase difference between the two orthogonal plasmon polarizabilities, which typically exhibits its maxima at wavelengths away from the peak of the longitudinal resonance and around the spectral overlap region of the longitudinal and the transverse resonances^19,39^. The excitation wavelength in our study is accordingly chosen at $\lambda = 633\,\hbox{nm}$, close to the peak of the longitudinal plasmon resonance. Therefore, in general, we observed both resonance-enhanced linear diattenuation and retardance effects at this excitation wavelength. Since the magnitude and the phases of the two orthogonal dipolar plasmon polarizabilities are influenced by the aspect ratio $\in$, changing $\in$ could also control and optimize the magnitudes of both diattenuation and retardance (Fig. 3a,b). In our case, we varied the GNRs length $l = 70\,\hbox{nm}$ to $105\,\hbox{nm}$ and diameter $d = 25\,\hbox{nm}$ to $71\,\hbox{nm}$ to change the aspect ratio by keeping the peak of the longitudinal resonance close to the excitation wavelength 633 nm. Specifically, for an aspect ratio $\in = 2.8$, maximum values of $D = 0.3$ and $R = 1.68$ were obtained through PSMC simulation, in excellent agreement with the experimental maximum values of $D = 0.295$ and $R = 1.71$. For value of aspect ratio $\in > 2.8$, diattenuation also shows linear behavior. However, control and tuning of the diattenuation and retardance effects by changing the aspect ratio of the GNRs with appropriate choice of the excitation wavelength thus appears to be an effective means of isolating the polarization effects of the GNRs from the corresponding background scattering effects of turbid media. Further, the diattenuation and retardance effects of the GNRs exhibit sharp spectral characteristic of the longitudinal and the transverse plasmon resonances, whereas those due to scattering from dielectrics typically exhibits broader wavelength variations.

Figure 3c,d show the dependence of linear diattenuation and retardance parameters on the concentrations of GNRs. Here the scattering coefficient due to the fixed concentration of microspheres was $\upmu_{S} = 102\,\hbox{cm}^{ - 1}$, whereas that induced by GNRs ranged from $\upmu_{S} = 16$ to $195\,\hbox{cm}^{ - 1}$. As noted above large magnitudes of linear diattenuation ($D = 0.287$) and retardance ($R = 1.69$ rad) corresponding to the lowest examined concentration of GNR likely arise due to a large difference in the amplitude and phases respectively of the two orthogonal resonant dipolar plasmon polarizabilities (excited by orthogonal linear polarizations).

Note that none of these intriguing trends were observed in the previous dielectric microspheres-only scenario (Fig. 2), where $D$ and $R$ did not show any appreciable variation with microsphere concentration. In contrast, here the magnitude of diattenuation and retardance is observed to *drop off* rapidly with increasing scattering coefficients (i.e., concentration) of GNRs. This interesting and somewhat counter-intuitive observation—GNR ‘anisotropic polarization signals’ getting weaker as their concentrations increases!—is likely due to the incoherent addition of the multiply scattered polarized light from the randomly oriented nanorods. As the size of the single GNR is very small compared to the focal spot size, the relative orientation of a large number of GNRs per unit volume (e.g., $2.4 \times 10^{7}$ particles per microliter for GNR-induced scattering coefficient of 180 cm^−1^) can add up constructively or destructively, leading to high or low anisotropic polarization signal. Evidently the latter effect (destructive superposition) dominates here, as D and R values diminish with the increasing GNR concentration, and thus the resultant randomness of their orientation/superposition increases. In our case, we have achieved diattenuation value $D = 0.28$ from the randomly oriented GNRs at the lowest studied value of nanorod-induced turbidity of $\upmu_{S} = 16\,\hbox{cm}^{ - 1}$, over and above $\upmu_{S} = 102\,\hbox{cm}^{ - 1}$ exhibited by the dielectric microspheres.

To put such GNR loadings into context, we note that typical concentrations of GNRs ranges from $\sim 25$ to 80 mg/ml for optical imaging in vivo^40^. However other methods such as computed tomography, photoacoustic and fluorescence imaging techniques typically report lower GNR concentrations (5–25  mg/ml)^40–44^. Our simulated and experimental results with GNR concentration range of $0.03$ to 2.5  mg/ml is typically $10$ to 100× lower than the *in-vivo* levels reported in the literature above. This is particularly promising as it demonstrates significant polarization contrast at rather modest GNR levels, at least in our controlled examined phantom system.

Following the linear diattenuation and retardance results, we now present the corresponding depolarization in Fig. 3e. Depolarization here appears to stem from (1) multiple scattering effects and (2) random orientation of the non-spherical metal nanorods. Both effects lead to incoherent addition of scattered polarized Stokes vectors of light (or incoherent addition of Mueller matrices of individual scatterers), eventually manifesting as net depolarization. However, unlike multiple scattering-induced depolarization from dielectric microspheres, the depolarization induced by scattering from the GNRs exhibit distinct spectral characteristics which strongly depends on their aspect ratios, previously reported by our group^19^. It is important to note that the wavelength corresponding to strongest depolarization does not coincide with the peak of linear diattenuation as shown in Supplementary Figure S1. Specifically, while the magnitude of diattenuation is maximum at wavelength corresponding to the peak of the longitudinal plasmon resonance of the GNR, the depolarization peak is observed at the overlap spectral region of the longitudinal and the transverse plasmon resonances, which is at a shorter wavelength. With increasing GNR aspect ratio, the depolarization peak further shifts spectrally away from the peak of the longitudinal resonance (and the wavelength peak of diattenuation also) towards shorter wavelength. Thus, with the choice of wavelength λ = 633 nm closer to the longitudinal plasmon resonance peak, one may obtain weak depolarization but strong diattenuation, even for randomized 3D orientation of GNRs, as is observed here.

Depolarization peaks at wavelengths away from the peak of the longitudinal resonance (− 633 nm) whereas diattenuation peak coincides with the longitudinal resonance^19^. Therefore, depolarization peaks at the overlapped wavelength regions of the longitudinal and the transverse resonance. Thus, it sees the diattenuation peak near the longitudinal resonance and is spectrally away from the depolarization peak (which is at shorter wavelength). Therefore, with increasing GNR aspect ratio, depolarization peak shifts away from the longitudinal resonance and towards shorter wavelength. As a result, even for highly randomized 3D orientation of GNRs, we observed maximum preservation of polarization (less depolarization) near the peak of the longitudinal plasmon resonance.

Figures 3e clearly demonstrate the preservation of polarization through PSMC simulations and experimentally in a GNRs + microspheres phantom system. Here, the *DOP* is plotted against varying GNR-induced scattering coefficients for a fixed microsphere scattering coefficient ($\upmu_{S} = 102\,\hbox{cm}^{ - 1}$). At the lowest GNR concentration examined ($\upmu_{S} = 16\,\hbox{cm}^{ - 1}$), depolarization reduced significantly compared to a microsphere-only system (cf. Fig. 2e): our theory and experiment yielded *DOP* values of ~ 70% compared to ~ 30% in the absence of GNRs at similar turbidity levels. This is particularly promising as a ‘polarization contrast enhancement effect’ in the biomedical context. As evident, one may obtain high magnitudes of polarization diattenuation and linear retardance (which act as the polarization contrast mechanism in this scenario) in the presence of considerably reduced depolarization effect with relatively small amount of added GNR as ‘contrast agent’. Thus, the exogenous in vivo additions of GNRs for polarization contrast may involve overall smaller amounts of injectable foreign substance, which is biomedically desirable. As expected, polarization loss scaled with increasing GNR concentrations, leading to *DOP* of $\sim 10\%$ at the highest medium turbidity examined ($\upmu_{S} = 195\,\hbox{cm}^{ - 1}$ due to GNRs, $\upmu_{S} = 102\,\hbox{cm}^{ - 1}$ due to microspheres). Yet significant polarization preservation is noted in the backscattering direction even in this very high scattering model system scenario.

As previously discussed, in addition to multiple scattering effects, the other prominent mechanism of depolarization in the composite heterogeneous system is the randomization of the scattering-induced phase retardance effects (between the two orthogonal dipolar plasmon polarizabilities) due to the random orientation of the GNRs. Overall then, judicious choices of the GNR concentrations, their aspect ratios and the choice of the wavelength will be key optimization parameters for contrast enhancement in polarimetric imaging of biological tissues.

## Conclusion

In this study, we examined polarization effects in optically turbid media containing polystyrene microspheres and GNRs, using both polarization sensitive Monte Carlo simulations and experimental Mueller matrix measurements. Backscattering geometry was chosen for both scientific and practical reasons (non-diminishing polarization signals and biological relevance, respectively). T-matrix formalism was incorporated into the existing polarization-sensitive Monte Carlo code to enable accurate modeling of GNR polarization effects. Parameters of the complex scattering medium—micro-and nano-particle sizes, shapes, concentrations—were systematically varied to quantify the resultant effects on medium polarization properties such as linear diattenuation, linear retardance, and depolarization. GNR-induced polarization preservation and increases in linear diattenuation and retardance were noted as a function of the various examined medium parameters. Importantly, significant GNR-enabled polarization contrast was observed despite their random 3D orientation. A notable finding was a *decrease* in GNR-induced polarization contrast effects with increasing nanorod concentration; in other words, GNR polarization contrast effects were more significant at generally *lower* nanorod concentrations. In all examined cases, simulation and experimental results showed excellent agreement, lending credence to the presented framework for examining model turbid media and showing promise for enabling GNR polarimetric studies in real biological tissues.

Large GNR-induced linear diattenuation and retardance values in the presence of large background turbidity suggest that these could be exploited for contrast enhancing mechanisms in polarimetric imaging of biological tissues. Specifically, diattenuation may prove easier and more amenable in clinical situations due to the ease of the measurement procedure; for example, one may directly obtain a tissue diattenuation image via a simple setup with suitable orientations of two linear polarizers in the excitation and detection paths. We are currently expanding our investigations towards *in-vivo* biomedical deployment of the proposed unique polarized light imaging strategy using GNRs for enhancing contrast in actual biological tissues, with potential applications in tumor margin detection, surgical guidance, and therapy monitoring.

## Materials and methods

Most used phantom systems to study the optical effects in turbid media are suspensions of solid scattering particles in a transparent background^44^. When the particles are of uniform size and shape and known refractive index (e.g., polystyrene microspheres and plasmonic GNRs in our study), the optical properties (e.g., scattering coefficient) and polarization effects (e.g., $\Delta ,R$ and $D$) can be calculated, the latter through Monte Carlo modelling. For our experiments, three different diameters of $0.42,0.65$ and $0.92\,\upmu \hbox{m}$ polystyrene microspheres (PM; Bangs Laboratories Inc.) suspension in water were used to mimic the turbidity of tissue, to which different concentrations of GNRs were then added. Mie theory for spherical scatterer was used to calculate the scattering cross-section of an individual microsphere σ, and suspensions with a precise number of microspheres per unit volume N were prepared to yield the desired medium scattering coefficient µ_s_ (via µ_s_ = σ · N). Therefore, we have prepared a stock solution of 0.1 (w/w) of polystyrene microspheres to get scattering coefficient $\upmu_{S} = 621\,\hbox{cm}^{ - 1}$. Sequential dilutions were performed by precisely adding distilled water aliquots to get the desired scattering coefficient down to $\upmu_{S} = 16\,\hbox{cm}^{ - 1}$. Similar process was followed for the $0.65\,\upmu \hbox{m}$ and $0.92\,\upmu \hbox{m}$ polystyrene microspheres. Then, the effects of the addition of GNRs were systematically investigated. For this purpose, we chose $d = 0.42\,\upmu \hbox{m}$ diameter microsphere suspension with $\upmu_{S} = 102\,\hbox{cm}^{ - 1}$ and added GNRs in 0.03 to 2.5 mg/ml concentration range. Keeping the microsphere suspension’s $\upmu_{S}$ fixed, appropriate addition of GNRs concentration and water were decided using the relation $C_{\% } (w/w) = m_{Solt} /m_{so\ln } *100$, here $C_{\% } (w/w)$,$m_{Solt}$ and $m_{so\ln }$ represents % concentration of the solid content, total mass of the solute (GNRs and microspheres) and mass of water respectively. We have considered the linear addition of the total mass of GNRs, PM and water to get the desired % concentration, thereafter the desired scattering coefficient. To ensure uniform distribution of added particles in the resultant suspensions, we sonicated each of the samples for 1 h, and the experiments were subsequently performed within 15 min of the preparation.

We have set up and calibrated the polarization Mueller matrix measurement system in the backscattering configuration as shown in Fig. 1. It consists of a 633 nm laser whose emission, after passing through a polarization state generator (PSG), was incident on a 50–50 beam splitter. The reflected light beam from the beam splitter was then focused on to the sample cuvette and the backscattered light from the sample was subsequently collected using a10 × microscope objective. The thickness of the cuvette was 1 cm and the width of its glass wall was ~ 1 mm. The incident laser beam diameter was 5 mm and after passing through the microscope objective, the diameter of the focal spot was ~ 1 mm. The backscattered light was collected by the same low-NA microscope objective, corresponding to an acceptance angle of ~ 10°. The collected backscattered light then passed through the beam splitter again and its polarization state was analyzed using a polarization state analyzer (PSA) and the polarization-resolved intensity signal was detected by a high-SNR photo detector. Each detected polarization-resolved intensity signal was recorded by taking average over 25 times recorded intensity signal. Thereafter, collecting 16 sequential polarization-resolved intensity measurement, Mueller matrix was constructed as discussed above.

The strategy for constructing the sample Mueller matrix in the backscattering geometry involves recording sixteen polarization-resolved intensity measurements for four different combinations of the optimized elliptical polarization generator (using PSG) and analyzer (using PSA) basis states. The PSG unit consists of a linear polarizer (*P1*) and a quarter wave plate (*Q1*) whereas a PSA unit consist of a quarter wave plate (*Q2*) and a linear polarizer (*P2*) placed in reverse order. Sequential changes of the fast axis of *Q1* to four angles (β = 35°, 70°, 105° and 140°) with respect to the axis of $P_{1}$ leads to generation of four optimized input elliptical polarization states (Stokes vectors)^42^. These four sets of Stokes vectors (each a 4 × 1 array) are then grouped as column vectors to form the 4 × 4 generator matrix $W$ for PSG. Likewise, the 4 × 4 analyzer matrix $A$ was formed by the four elliptical polarization basis states obtained by changing the fast axis of $Q_{2}$ to the same four angles (β = 35°, 70°, 105° and 140°). The sixteen sequential intensity measurements were grouped in a 4 × 4 measurement matrix $M_{i}$ which is related to $A$*, *$W$ and the sample Mueller matrix $M$ as $M_{i} = AMW$. The sample Mueller matrix $M$ was then determined from the known forms of $A$ and $W$ matrices via $M = A^{ - 1} M_{i} W^{ - 1}$. Details on the optimization of the polarization basis states in the PSG and the PSA matrices, including optimization of the orientation angle of the quarter waveplates with respect to the polarizers, have been published^45^.

In principle, Mueller matrix $M$ can be determined from experimental $M_{i}$, by using theoretical forms of $A$ and $W$ metrices (obtained by using the standard Mueller metrices of the polarizer and the quarter wave plates). However, this is confounded by the complex nature of the polarization effects caused by non-ideal behavior of polarization optical elements, slight misalignments, excitation/collection/focusing geometry details and so forth. These can lead to significant deviations in the actual experimentally determined $W$ and $A$ metrices from their ideal theoretical forms. We have taken care of these issues through a robust eigenvalue calibration method (ECM) yielding accurate information of the polarization response of the PSG and the PSA units by determining the exact experimental forms of the $W$ and $A$ matrices^45^. Using this approach, $W$ and $A$ are determined by performing measurements on ideal calibration samples with known polarization properties, such as pure attenuators (polarizers) and retarders (wave plates). The ensuing calibration results are presented in Fig. 1, demonstrating good $MM$ polarimeter performance and enabling robust and accurate measurements in the complex turbid media as described above.

## Supplementary Information

Supplementary Information.
