# Supplementary material for: Diattenuation and retardance signature of plasmonic gold nanorods in turbid media revealed by Mueller matrix polarimetry
Source: Sci Rep. 2021 Oct 8;11:20017. doi: 10.1038/s41598-021-99430-6 (PMC8501131; doi:10.1038/s41598-021-99430-6)
Supplement: Supplementary file 1 — Supplementary Information. [file 41598_2021_99430_MOESM1_ESM.docx]

Supplementary document

**Diattenuation and retardance signature of plasmonic gold nanorods in turbid media revealed by Mueller matrix polarimetry**

Subir Kumar Ray^1,2*^, Nirmalya Ghosh^3^, Alex Vitkin^1,2,4*^

^1^Division of Biophysics and Bioimaging, Princess Margaret Research Institute, Toronto, Canada,

^2^Department of Medical Biophysics, University of Toronto, Toronto, Canada

^3^Indian Institute of Science Education and Research (IISER) Kolkata, Mohanpur, 741246, India

^4^Department of Radiation Oncology, University of Toronto, Toronto, Canada

1. **Generalized T-matrix formalism.**

In a T-matrix framework, the incident and the scattered fields from a long cylindrical scatterer (length >> diameter) can be expressed as [1]

$\left( \begin{matrix} E_{\parallel s} \\ E_{\perp s} \end{matrix} \right)=e^{{i3\pi}/4}\sqrt{\frac{2}{\pi kr\sin\xi}}e^{ik(r\sin\xi-z\cos\xi)}\left( \begin{matrix} T^{11} & T^{12} \\ T^{21} & T^{22} \end{matrix} \right)\left( \begin{matrix} E_{\parallel i} \\ E_{\perp i} \end{matrix} \right)$ (S1)

where the T-matrix elements are

$T^{11}=\sum_{-\infty}^{+\infty} b_{\mathrm{nI}}e^{-in\theta}$; $T^{12}=\sum_{-\infty}^{+\infty} a_{\mathrm{nII}}e^{-in\theta}$;

$T^{21}=\sum_{-\infty}^{+\infty} a_{\mathrm{nI}}e^{-in\theta}$; $T^{22}=\sum_{-\infty}^{+\infty} b_{\mathrm{nII}}e^{-in\theta}$;

And the coefficients are

$a_{\mathrm{nI}}=\frac{C_{n}V_{n}-B_{n}D_{n}}{w_{n}V_{N}+iD_{n}^{2}}$ $a_{\mathrm{nII}}=\frac{A_{n}V_{n}-C_{n}D_{n}}{w_{n}V_{N}+iD_{n}^{2}}$ ; $B_{\mathrm{nI}}=\frac{W_{n}B_{n}+iD_{n}C_{n}}{w_{n}V_{N}+iD_{n}^{2}}$ ; $B_{\mathrm{nII}}=\frac{{-iC}_{n}W_{n}+A_{n}D_{n}}{w_{n}V_{N}+iD_{n}^{2}}$

where $A_{n}=i\zeta[\zeta J_{n}^{'}\left( n \right)J_{n}\left( \zeta\right)-\eta J_{n}\left( \eta\right)J_{n}^{'}\left( \zeta\right)]$ ; $B_{n}=\zeta[m^{2}\zeta J_{n}^{'}\left( n \right)J_{n}\left( \zeta\right)-\eta J_{n}\left( \eta\right)J_{n}^{'}\left( \zeta\right)]$

$C_{n}=ncos\zeta nJ_{n}\left( \zeta\right)J_{n}(\zeta)\left( \frac{\zeta^{2}}{\eta^{2}}-1 \right)$ ; $D_{n}=ncos\zeta nJ_{n}\left( \zeta\right)H_{n}^{1}(\zeta)\left( \frac{\zeta^{2}}{\eta^{2}}-1 \right)$

$W_{n}=i\zeta[\eta J_{n}^{'}\left( \eta\right)H_{n}^{1'}\left( \zeta\right)-\zeta J_{n}^{'}\left( \eta\right)H_{n}^{1}(\zeta)]$; $V_{n}=\zeta[m^{2}{\zeta J}_{n}^{'}\left( \eta\right)H_{n}^{1}\left( \zeta\right)-\eta J_{n}\left( \eta\right)H_{n}^{1'}(\zeta)]$

$\zeta=x\sin\zeta$ ; $\eta=x\sqrt{\left( m^{2}-\cos^{2}\zeta\right)}$ ; $x=ka$

$J_{n}$ is Bessel function; $H_{n}^{1}=J_{n}+iY_{n}$ is the Hankel function.

Now consider a finite scattering object occupying a region $V_{\mathrm{INT}}$ bounded by a closed surface $\mathbf{S}$ and imbedded in an infinitely extended homogeneous, isotropic, nonmagnetic and non-absorbing medium. The infinite region exterior to the particle is denoted by $V_{\mathrm{EXT}}$. The electric field for $r^{'}\in V_{\mathrm{EXT}}$ can be written as [1-2]

$\mathbf{E}\left( r \right)=\mathbf{E}^{\mathrm{inc}}\left( \mathbf{r}^{\mathbf{'}} \right)+\mathbf{E}^{\mathrm{Scat}}\left( \mathbf{r}^{'} \right)$ (S2)

The known incident electric field can be express in terms of spherical harmonics as [1-2]

$\mathbf{E}^{\mathrm{inc}}\left( \mathbf{r}^{\mathbf{'}} \right)=\sum_{n=1}^{\infty} \sum_{m=-n}^{n} \left[ a_{\mathrm{mn}}\mathrm{Rg}\mathbf{M}_{\mathrm{mn}}\left( k_{1}\mathbf{r}' \right)+b_{\mathrm{mn}}\mathrm{Rg}\mathbf{N}_{\mathrm{mn}}\left( k_{1}\mathbf{r}' \right) \right]$ (S3)

where $a_{\mathrm{mn}}=k_{1}\left( -1 \right)^{m}\int\mathrm{ds}\left\{ \omega\mu_{0}\left[ \left( \hat{\mathbf{n}}\times\mathbf{H}_{+}\left( \mathbf{r} \right) \right) \right]\mathbf{M}_{-mn}\left( k_{1}r,\theta,\varphi\right)-ik_{1}\left[ \left( \hat{\mathbf{n}}\times\mathbf{E}_{+}\left( r \right) \right) \right]\mathbf{N}_{-mn}\left( k_{1}r,\theta,\varphi\right) \right\}$ (S4)

and $b_{\mathrm{mn}}=k_{1}\left( -1 \right)^{m}\int\mathrm{ds}\left\{ \omega\mu_{0}\left[ \left( \hat{\mathbf{n}}\times\mathbf{H}_{\mathbf{+}}\left( r \right) \right) \right]\mathbf{N}_{-mn}\left( k_{1}r,\theta,\varphi\right)-ik_{1}\left[ \left( \hat{\mathbf{n}}\times\mathbf{E}_{\mathbf{+}}\left( r \right) \right) \right]\mathbf{M}_{-mn}\left( k_{1}r,\theta,\varphi\right) \right\}$ (S5)

Similarly, the scattered electric field can be express in terms of spherical harmonics as

$\mathbf{E}^{\mathrm{Scat}}\left( \mathbf{r}^{\mathbf{'}} \right)=\sum_{n=1}^{\infty} \sum_{m=-n}^{n} \left[ p_{\mathrm{mn}}\mathbf{M}_{\mathrm{mn}}\left( k_{1}\mathbf{r}' \right)+q_{\mathrm{mn}}\mathbf{N}_{\mathrm{mn}}\left( k_{1}\mathbf{r}' \right) \right]$ (S6)

where

$p_{\mathrm{mn}}={-k}_{1}\left( -1 \right)^{m}\int\mathrm{ds}\left\{ \omega\mu_{0}\left[ \left( \hat{\mathbf{n}}\times\mathbf{H}_{+}\left( \mathbf{r} \right) \right) \right]{\mathrm{Rg}\mathbf{M}}_{-mn}\left( k_{1}r,\theta,\varphi\right)-ik_{1}\left[ \left( \hat{\mathbf{n}}\times\mathbf{E}_{+}\left( \mathbf{r} \right) \right) \right]{\mathrm{Rg}\mathbf{N}}_{-mn}\left( k_{1}r,\theta,\varphi\right) \right\}$ (S7)

$q_{\mathrm{mn}}={-k}_{1}\left( -1 \right)^{m}\int\mathrm{ds}\left\{ \omega\mu_{0}\left[ \left( \hat{\mathbf{n}}\times\mathbf{H}_{+}\left( \mathbf{r} \right) \right) \right]{\mathrm{Rg}\mathbf{N}}_{-mn}\left( k_{1}r,\theta,\varphi\right)-ik_{1}\left[ \left( \hat{\mathbf{n}}\times\mathbf{E}_{+}\left( \mathbf{r} \right) \right) \right]{\mathrm{Rg}\mathbf{M}}_{-mn}\left( k_{1}r,\theta,\varphi\right) \right\}$ (S8)

The expansion coefficients of the scattered fields in Eqs. S7 and S8 can be calculated by assuming the electric field inside the scattering object can be expressed through regular vector spherical wave functions of the interior wave equation that can be obtained from Eqs. S4 and S5 as

$\mathbf{E}\left( \mathbf{r} \right)=\sum_{n'=1}^{\infty} \sum_{m'=-n'}^{n'} \left[ C_{m'n'}\mathrm{Rg}\mathbf{M}_{m'n'}\left( k_{2}\mathbf{r} \right)+D_{m'n'}\mathrm{Rg}\mathbf{N}_{m'n'}\left( k_{2}\mathbf{r} \right) \right] r\in V_{\mathrm{INT}}$ ; (S9)

with $k_{2}$ being the wave number in the interior region. And the magnetic field expansion becomes,

$\mathbf{H}\left( \mathbf{r} \right)=\frac{k_{2}}{i\omega\mu_{0}}\sum_{n'=1}^{\infty} \sum_{m'=-n'}^{n'} \left[ D_{m'n'}\mathrm{Rg}\mathbf{M}_{m'n'}\left( k_{2}\mathbf{r} \right)+C_{m'n'}\mathrm{Rg}\mathbf{N}_{m'n'}\left( k_{2}\mathbf{r} \right) \right] r\in V_{\mathrm{INT}}$ (S10)

Using continuity of tangential components of the electromagnetic fields

$\hat{\mathbf{n}}\times\mathbf{E}_{+}\left( \mathbf{r} \right)=\hat{\mathbf{n}}\times\mathbf{E}_{-}\left( \mathbf{r} \right)$

$r\in S$ (S11)

$$\hat{\mathbf{n}}\times\mathbf{H}_{+}\left( \mathbf{r} \right)=\hat{\mathbf{n}}\times\mathbf{H}_{-}\left( \mathbf{r} \right)$$

and substituting Eqs. S9, S10 and S11 into Eqs. S4 and S5 yields

$\left[ \begin{matrix} a \\ b \end{matrix} \right]=\left[ \begin{matrix} Q^{11} & Q^{12} \\ Q^{21} & Q^{22} \end{matrix} \right]\left[ \begin{matrix} c \\ d \end{matrix} \right]$ (S12)

Where

$$Q_{\mathrm{mn}m^{'}n^{'}}^{11}=-ik_{1}k_{2}J_{\mathrm{mn}{m^{'}n}^{'}}^{21}-ik_{1}^{2}J_{\mathrm{mn}{m^{'}n}^{'}}^{12}$$

$$Q_{\mathrm{mn}m^{'}n^{'}}^{12}=-ik_{1}k_{2}J_{\mathrm{mn}{m^{'}n}^{'}}^{11}-ik_{1}^{2}J_{\mathrm{mn}{m^{'}n}^{'}}^{22}$$

$$Q_{\mathrm{mn}m^{'}n^{'}}^{21}=-ik_{1}k_{2}J_{\mathrm{mn}{m^{'}n}^{'}}^{22}-ik_{1}^{2}J_{\mathrm{mn}{m^{'}n}^{'}}^{11}$$

$$Q_{\mathrm{mn}m^{'}n^{'}}^{22}=-ik_{1}k_{2}J_{\mathrm{mn}{m^{'}n}^{'}}^{12}-ik_{1}^{2}J_{\mathrm{mn}{m^{'}n}^{'}}^{21}$$

And

$\left[ \begin{matrix} \begin{matrix} J_{\mathrm{mn}{m^{'}n}^{'}}^{11} \\ J_{\mathrm{mn}{m^{'}n}^{'}}^{12} \end{matrix} \\ \begin{matrix} J_{\mathrm{mn}{m^{'}n}^{'}}^{21} \\ J_{\mathrm{mn}{m^{'}n}^{'}}^{22} \end{matrix} \end{matrix} \right]=\left( -1 \right)^{m}\int\mathrm{ds}\hat{\mathbf{n}}\mathbf{.}\left[ \begin{matrix} \begin{matrix} \mathrm{Rg}\mathbf{M}_{m'n'}\left( k_{2}\mathbf{r},\theta,\varphi\right)\times\mathbf{M}_{-mn}\left( k_{1}\mathbf{r},\theta,\varphi\right) \\ \mathrm{Rg}\mathbf{M}_{m'n'}\left( k_{2}\mathbf{r},\theta,\varphi\right)\times\mathbf{N}_{-mn}\left( k_{2}\mathbf{r},\theta,\varphi\right) \end{matrix} \\ \begin{matrix} \mathrm{Rg}\mathbf{N}_{m'n'}\left( k_{2}\mathbf{r},\theta,\varphi\right)\times\mathbf{M}_{-mn}\left( k_{2}\mathbf{r},\theta,\varphi\right) \\ \mathrm{Rg}\mathbf{N}_{m'n'}\left( k_{2}\mathbf{r},\theta,\varphi\right)\times\mathbf{N}_{-mn}\left( k_{2}\mathbf{r},\theta,\varphi\right) \end{matrix} \end{matrix} \right]$

Here, a and b represent the expansion coefficients of the incident plane waves; p and q represent the expansion coefficients of the scattered fields; c and d represent the expansion coefficients of the internal fields.

Similarly, substituting Eqs. S9, S10 and S11 into Eqs. S7 and S8) we get

$\left[ \begin{matrix} p \\ q \end{matrix} \right]=-\left[ \begin{matrix} {\mathrm{Rg}Q}^{11} & {\mathrm{Rg}Q}^{12} \\ \mathrm{Rg}Q^{21} & {\mathrm{Rg}Q}^{22} \end{matrix} \right]\left[ \begin{matrix} c \\ d \end{matrix} \right]$ (S13)

where , $\mathrm{Rg}Q_{\mathrm{mn}m^{'}n^{'}}^{11}=-ik_{1}k_{2}\mathrm{Rg}J_{\mathrm{mn}{m^{'}n}^{'}}^{21}-ik_{1}^{2}\mathrm{RgJ}_{\mathrm{mn}{m^{'}n}^{'}}^{12}$

$\mathrm{RgQ}_{\mathrm{mn}m^{'}n^{'}}^{12}=-ik_{1}k_{2}\mathrm{Rg}J_{\mathrm{mn}{m^{'}n}^{'}}^{11}-ik_{1}^{2}\mathrm{Rg}J_{\mathrm{mn}{m^{'}n}^{'}}^{22}$

$\mathrm{RgQ}_{\mathrm{mn}m^{'}n^{'}}^{21}=-ik_{1}k_{2}\mathrm{Rg}J_{\mathrm{mn}{m^{'}n}^{'}}^{22}-ik_{1}^{2}\mathrm{RgJ}_{\mathrm{mn}{m^{'}n}^{'}}^{11}$

$\mathrm{RgQ}_{\mathrm{mn}m^{'}n^{'}}^{22}=-ik_{1}k_{2}\mathrm{Rg}J_{\mathrm{mn}{m^{'}n}^{'}}^{12}-ik_{1}^{2}\mathrm{RgJ}_{\mathrm{mn}{m^{'}n}^{'}}^{21}$

and

$$\left[ \begin{matrix} \begin{matrix} {\mathrm{Rg}J}_{\mathrm{mn}{m^{'}n}^{'}}^{11} \\ \mathrm{Rg}J_{\mathrm{mn}{m^{'}n}^{'}}^{12} \end{matrix} \\ \begin{matrix} {\mathrm{Rg}J}_{\mathrm{mn}{m^{'}n}^{'}}^{21} \\ \mathrm{Rg}J_{\mathrm{mn}{m^{'}n}^{'}}^{22} \end{matrix} \end{matrix} \right]=\left( -1 \right)^{m}\int\mathrm{ds}\hat{\mathbf{n.}}\left[ \begin{matrix} \begin{matrix} \mathrm{Rg}\mathbf{M}_{m'n'}\left( k_{2}\mathbf{r},\theta,\varphi\right)\times{\mathrm{Rg}\mathbf{M}}_{-mn}\left( k_{1}\mathbf{r},\theta,\varphi\right) \\ \mathrm{Rg}\mathbf{M}_{m'n'}\left( k_{2}\mathbf{r},\theta,\varphi\right)\times Rg\mathbf{N}_{-mn}\left( k_{2}\mathbf{r},\theta,\varphi\right) \end{matrix} \\ \begin{matrix} \mathrm{Rg}\mathbf{N}_{m'n'}\left( k_{2}\mathbf{r},\theta,\varphi\right)\times{\mathrm{Rg}\mathbf{M}}_{-mn}\left( k_{2}\mathbf{r},\theta,\varphi\right) \\ \mathrm{Rg}\mathbf{N}_{m'n'}\left( k_{2}\mathbf{r},\theta,\varphi\right)\times{\mathrm{Rg}\mathbf{N}}_{-mn}\left( k_{2}\mathbf{r},\theta,\varphi\right) \end{matrix} \end{matrix} \right]$$

Comparing Eq. 3A, Eq. S12 and Eq. S13 we get

$T^{\mathrm{ij}}=-(RgQ_{mnm'n'}^{\mathrm{ij}}(kr,\vartheta,\varphi)){Q_{mnm'n'}^{\mathrm{ij}}(kr,\vartheta,\varphi)}^{-1}$ (S14)

Here, $Q_{\mathrm{mn}}\left( kr,\vartheta,\varphi\right)=h_{n}^{1}(kr)({-1)}^{m}\sqrt{{\left( n+m \right)!}/{\left( n-m \right)!}}d_{0m}^{n}\left( \vartheta\right)e^{im\varphi}$ and $\mathrm{Rg}Q_{\mathrm{mn}}\left( kr,\vartheta,\varphi\right)=j_{n}(kr)({-1)}^{m}\sqrt{{\left( n+m \right)!}/{\left( n-m \right)!}}d_{0m}^{n}(\vartheta)e^{im\varphi}$; $j_{n}$&$d_{0m}^{n}$are Bessel and Wigner d-functions functions with $n=0,1,2,\ldots; m=0, \pm1, \pm2, \ldots.$.$Q_{\mathrm{mn}m^{'}n^{'}}^{\mathrm{ij}}$ were calculated using numerical integral $J_{\mathrm{mn}{m^{'}n}^{'}}^{\mathrm{ij}}$of the matrix elements as [3]:

For a rotationally symmetric scattering particle (such as a GNR), with the z-axis of the particle reference frame along the axis of rotation, radius vector can be expressed as of

$r\left( \theta,\varphi\right)=r(\vartheta)\hat{\mathbf{r}}$ and $\mathrm{ds}\hat{\mathbf{n}}(r)\mathbf{=}\mathbf{r}^{\mathbf{2}}\sin\vartheta\left( \hat{\mathbf{r}}\mathbf{-}\frac{\mathbf{r}_{\boldsymbol{\vartheta}}}{\mathbf{r}}\hat{\boldsymbol{\vartheta}} \right)\mathbf{d}\vartheta d\varphi$ (S14A)

However, in our experimental case we have used a dog bone shaped GNR whose geometrical shape is defined by five parameters: the length $l$, central diameter $d$, end cape diameter $d_{1}=d(1+\chi_{d})$, elliptical end-cap thickness $b_{1}=\chi_{1}(d_{1}/2)$, and the shape of the generating line between the endcaps. Generating line is defined by the x-shifted circle $\left( x-b-R \right)^{2}+z^{2}=R^{2}$ . Using this approach, the T-matrix elements of an axially symmetric non-ideal finite GNRs (like the ones used in our study) can be determined from the known incident electric field. For a finite GNR with a radius vector *r*$\left( \boldsymbol{\vartheta} \right)$, components of the T matrix can be calculated from the interior scattered field component ($Q_{\mathrm{mn}m^{'}n^{'}}^{\mathrm{ij}}\left( kr^{'},\vartheta,\varphi\right)$for $r^{'}<r$) and ($\mathrm{Rg}Q_{mnm'n'}^{\mathrm{ij}}(kr^{'},\vartheta,\varphi)$ for $r^{'}>r$) through the expansion of scattered electric fields using the integral

$J_{\mathrm{mn}{m^{'}n}^{' =}}^{\mathrm{ij}}\int_{-1}^{1} d\left( \cos\vartheta\right)F_{\mathrm{mn}{m^{'}n}^{'}}^{\mathrm{ij}}\left( \vartheta,k\sqrt{\varepsilon_{m}}r\left( \vartheta\right),k\sqrt{\varepsilon}r\left( \vartheta\right),r'\left( \vartheta\right) \right)$ (S15)

where $i,j=1,2; -n\leq m\leq n;n=1,2,\ldots$;$k\sqrt{\varepsilon_{m}}$ and $k\sqrt{\varepsilon}$ are the wave numbers in the external medium and inside the GNRs$,$the radius vector $r\left( \vartheta\right)$defines the shape of the GNRs as a function of polar angle which incorporates central diameter, end-cap diameter, end-cap elliptical thickness and the shape of the generating line between the end caps; the function $F_{\mathrm{mn}{m^{'}n}^{'}}^{\mathrm{ij}}$is defined through the vector spherical harmonics and its derivatives [3]. Note that to incorporate the above model, the standard T matrix code [1-3] has been modified to determine the radius vector $r_{1}(\vartheta) and r_{2}(\vartheta)$ and their logarithmic derivatives accordingly.

1. **Polar Decomposition**

To get quantitative polarization information from the experimentally obtained or theoretically derived Mueller metrices, further mathematics are required. This is because the various medium polarization properties (such as diattenuation, retardance, depolarization, etc) are admixed in the 16 elements of the matrix, and as such additional analysis is required for their unambiguous extraction. Lu and Chipman described one popular approach known as the Polar Decomposition Method (PDM) for extracting three sequential polarization basis metrices that isolate individual polarization properties: deattenuating matrix $M_{d}$, retarding matrix $M_{R}$ and depolarizing matrix $M_{\Delta}$ [4]

$M\Longleftarrow M_{\Delta}.M_{R}.M_{D}$ (S16)

where$M_{D}=\left( \begin{matrix} 1 & \mathbf{D} \\ \mathbf{D} & \mathbf{m}_{\mathbf{D}} \end{matrix} \right), M_{\Delta}=\left( \begin{matrix} 1 & \mathbf{0}^{\mathbf{T}} \\ P_{\Delta} & m_{\delta} \end{matrix} \right), M_{R}=\left( \begin{matrix} 1 & \mathbf{0}^{\mathbf{T}} \\ 0 & m_{R} \end{matrix} \right)$ and $\mathbf{D}=\frac{1}{M_{11}}\left[ M_{12}M_{13}M_{14} \right]^{T}$ is the deattenuating vector. Then to derive actual material property values from these basis sub-metrices of the original Mueller matrix, we further analyze these three. Specifically, the magnitude of

$\left| \mathbf{D} \right|=\frac{1}{M_{11}}\sqrt{M_{12}^{2}+M_{13}^{2}+M_{14}^{2}}$ (S17)

gives the total diattenuation of the system. This is what we calculate and plot in Figures 2 and 3 of the manuscript. Similarly, $\mathbf{P}=\frac{1}{M_{11}}\left[ M_{21}M_{31}M_{41} \right]^{T}$ is the polarizance vector. $m_{\delta}$ and $m_{R}$ are 3 x 3 sub-metrices of $M_{\Delta}$and $M_{R}$ and can be found by solving the eigenvalues of $m^{'}=m_{\delta}.m_{R}$. The net retardance can be extracted as $R=\cos^{-1} \left( \frac{Tr(M_{R})}{2}-1 \right)$ and the linear retardance as

$\delta=\cos^{-1} \left[ \sqrt{{{(M}_{R,22}+M_{R,33})}^{2}+{{(M}_{R,32}+M_{R,23})}^{2}}-1 \right]$ (S18)

Similarly, the net depolarization coefficient *$\Delta$* can be extracted as

$\Delta=1-\frac{\left|tr(M_{\Delta}-1)\right|}{3},0\leq\Delta\leq1$ (S19)

Figure 2(e) and 3(e) of the manuscript shows the plot for degree of polarization (DOP), which is $\frac{\left| tr(M_{\Delta}-1) \right|}{3}$.

Note also that although we employ the commonly used PDM variant for MM analysis and property extraction in this work, there are alternative decomposition methods. Their advantages and disadvantages have been extensively discussed in the literature [4-5], and active research in this field continues [6-7]

1. **Spectral dependence of depolarization**

T-matrix computation method was used to calculate the scattering efficiency as a function of wavelength shown in Figure S1(a). The computation was carried out for a single dog boned shaped nanorod with length $l=70 nm$ ; central diameter $d=25 nm$; end cap diameter $d_{1}=30 nm$. Two distinct resonance modes observed at 520 nm (TM-mode) and 640 nm (TE-mode). Figure S1(b) shows the T-matrix computed and Muller matrix derived depolarization (Δ) as a function of wavelength. Interestingly, depolarization peaks near the overlap region between the TE- and TM-modes. Thus, figure S1 justify our discussion in proper choice of excitation wavelength as 633 nm.


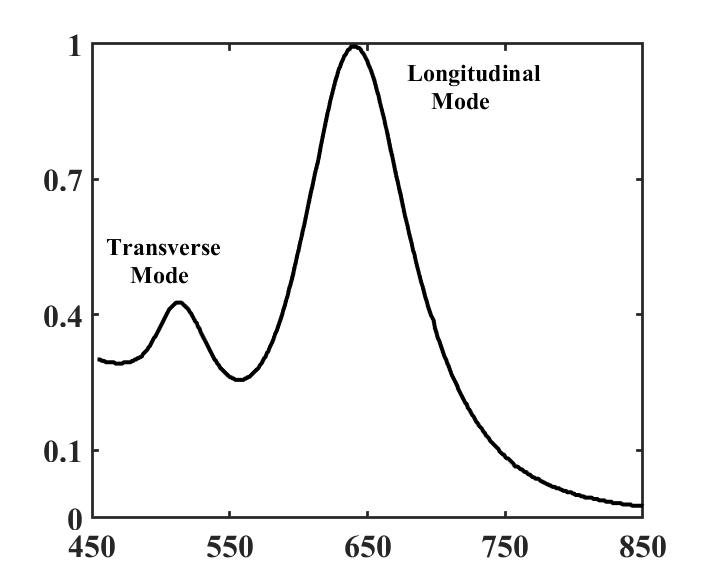

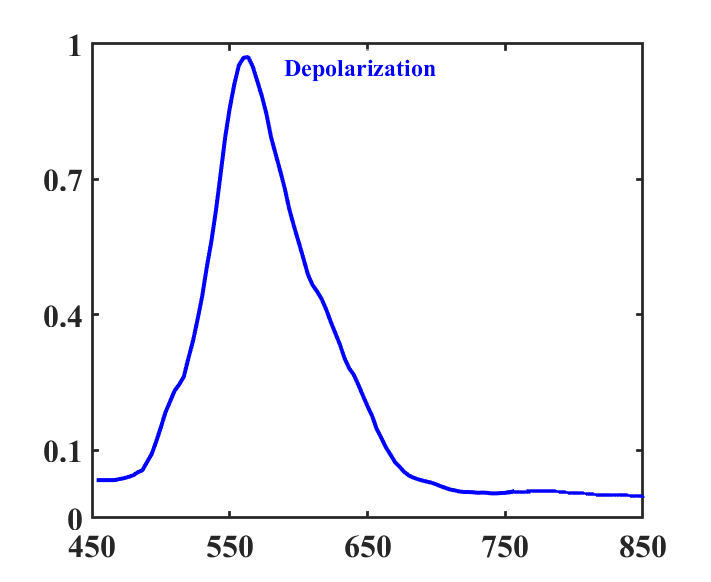


**Wavelength (λ) in nm**

**Wavelength (λ) in nm**

**Normalized intensity**

**Normalized intensity**

**a)**

**b)**

**Figure S1:** Simulated wavelength dependency of localized surface plasmon resonance (LSPR) modes (a) and depolarization (b). Dimensions chosen for GNR are length=70 nm and average diameter=25 nm. Depolarization has maxima near 570 nm which is far away from the longitudinal LSPR mode at 640 nm

**Reference:**

1. Bohren, C.F. and Huffman, D.R.  *Absorption and scattering of light by small particles*. John Wiley &Sons. (2008).
2. Mishchenko, Michael I., Larry D. Travis, and Andrew A. Lacis. Scattering, absorption, and emission of light by small particles. Cambridge university press. (2002).
3. Mishchenko, M.I. and Travis, L.D. Capabilities and limitations of a current FORTRAN implementation of the T-matrix method for randomly oriented, rotationally symmetric scatterers. Journal of Quantitative Spectroscopy and Radiative Transfer, **60**, 309-324,(1998).
4. Lu, S.Y. and Chipman, R.A. Interpretation of Mueller matrices based on polar decomposition. *JOSA A*, **13**, 1106-1113, (1996).
5. Ortega-Quijano, N. and Arce-Diego, J.L. Mueller matrix differential decomposition. *Optics letters*, *36*(10), 1942-1944 (2011).
6. Singh, G. and Yamaguchi, Y. Model-based six-component scattering matrix power decomposition. *IEEE Transactions on Geoscience and Remote Sensing*, *56*(10), 5687-5704 (2018).
7. Li, H., Zhao, Z., Zhou, X. and Ren, K. Mueller matrix decompositions of depolarizing medium based on eigenvalues of coherency matrices. *Optik*, *207*, 163799, (2020).
